# Increasing Incidence of Coronary Artery Disease in Younger Men: Case Study of a 39-Year-Old

**DOI:** 10.7759/cureus.74516

**Published:** 2024-11-26

**Authors:** Jad Elgaali, Patrick Coello de la Cruz, Syed I Hussaini, Mehjabeen Naseer

**Affiliations:** 1 Clinical Department, Saint James School of Medicine, The Quarter, AIA; 2 Medicine, Faculty of Medicine and Health Sciences, Catholic University of Valencia, San Vicente Mártir, Valencia, ESP; 3 Internal Medicine, Insight Hospital and Medical Center, Chicago, USA; 4 Internal Medicine, Swedish Covenant Hospital, Chicago, USA

**Keywords:** adult cardiology, coronary artery disease, left anterior descending stenosis, significant coronary artery disease, single vessel disease

## Abstract

Coronary artery disease (CAD) is the leading cause of death globally. While it is usually diagnosed after years of declining health or after a myocardial infarction (MI), we found that patients can be asymptomatic, posing a latent and life-threatening risk. We present the case of a man less than 40 years old, in whom heart disease was incidentally discovered during a CT scan performed for an unrelated condition.

We examine the most common causes of CAD, the pathophysiology and discuss potential methods to screen for it before patients are in need of urgent or emergent interventional care due to an MI or other ischemic events. We also discuss the risk factors, many of which are modifiable, as well as the financial burden CAD places on the healthcare system and the patient. This case report contributes to the body of knowledge in the field of cardiology by bringing forward and emphasizing that CAD is no longer solely a disease of the elderly and that we need to begin rethinking how and when we screen for the leading cause of death in the world.

## Introduction

Coronary vascular disease (CVD) remains the leading cause of death worldwide, accounting for almost one-third of total deaths globally [1,2]. The most common subtype of CVD is coronary artery disease (CAD), a disease that affects the arteries. Although deaths due to CAD peaked in the 1960s, it remains the most prevalent cause of death today, accounting for 85% of CVD deaths, most commonly due to myocardial infarction (MI) [1,2]. The pathophysiology of CAD involves the formation of what is commonly referred to as “plaque,” which is a build up of lipids. Over time, it grows over the lumen of the arteries and impedes blood flow [2]. The first step in atherosclerosis is endothelial dysfunction caused by a variety of stressors such as hypertension, high cholesterol, hyperglycemia (in the form of either diabetes or metabolic syndrome), and smoking [3]. Most of these stressors are interlinked, often causing and/or accentuating one another. These stressors lead to the recruitment of inflammatory cells such as macrophages and platelets which secretes molecules such as platelet derived growth factor (PDGF) and PDGF subsequently stimulates smooth muscle cells. Smooth muscle cells and macrophages consume cholesterol and lipids in the form of oxidized low-density lipoprotein (LDL) and create foam cells which form fatty streaks. These fatty streaks become the base layer of the early atheroma upon which plaque builds over the course of decades and ultimately causes CAD [2]. 

## Case presentation

**Figure 1:**
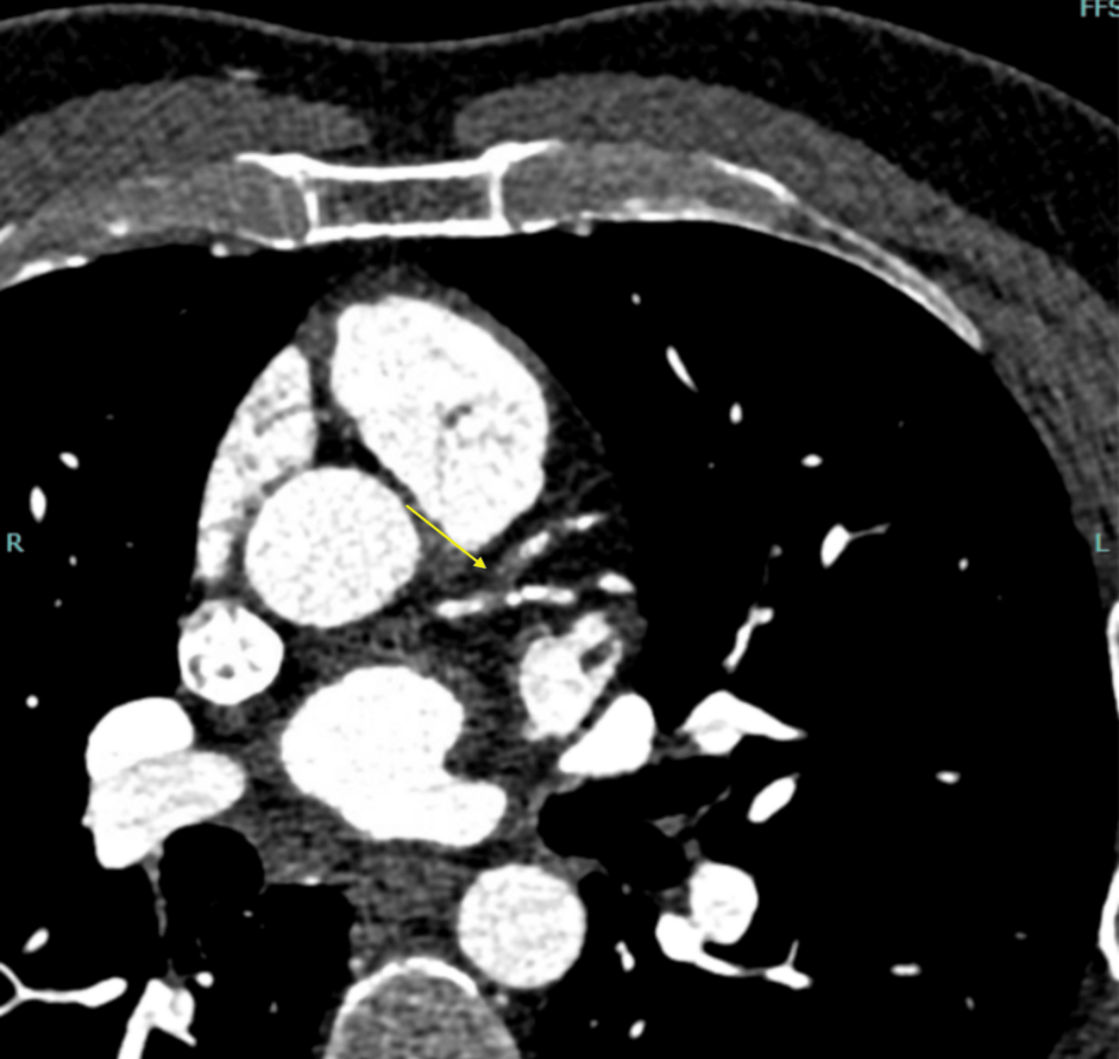
CTA demonstrating stenosis of the LAD artery (yellow arrow) CTA: CT angiogram; LAD: Left anterior descending

**Figure 2:**
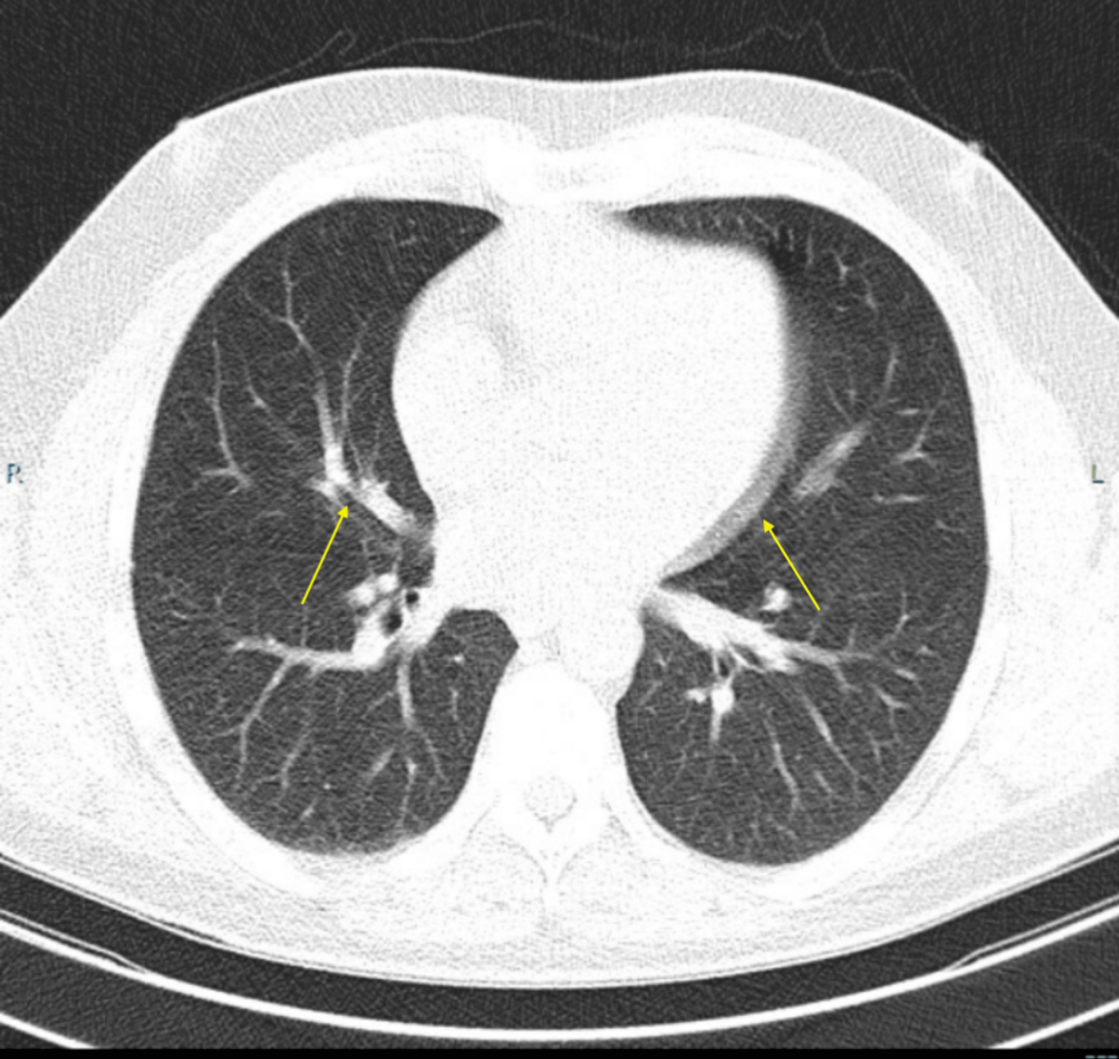
Low-dose CT demonstrating epicardial and coronary artery calcifications The yellow arrow on the right in points to the epicardium, which showed calcifications, and the one on the left points to coronary atherosclerosis.

**Figure 3:**
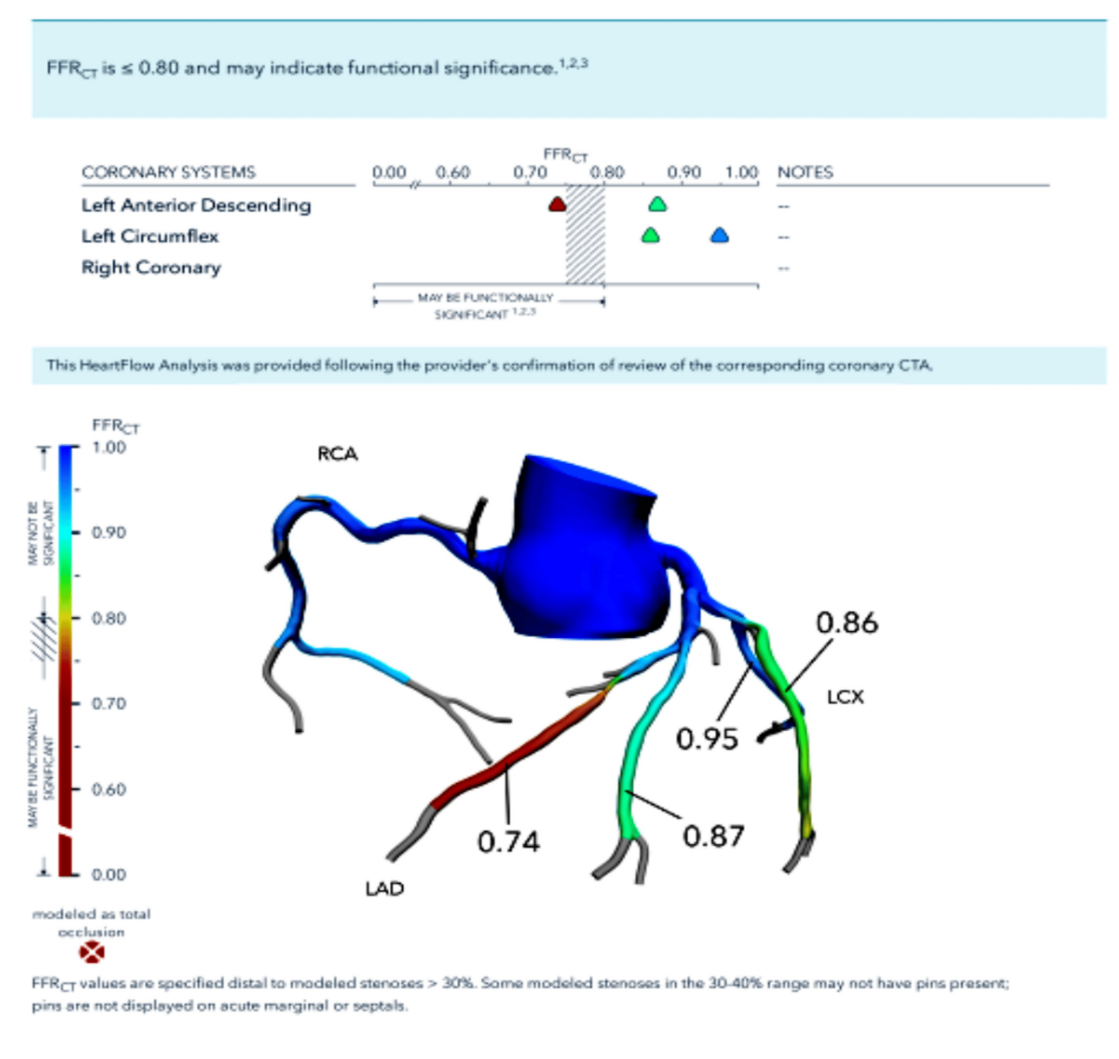
HeartFlow® analysis shows 99% stenosis of the LAD artery after the diagonal bifurcation indicated by the red vessel LAD: Left anterior descending

An asymptomatic 39-year-old male had repeatedly tested positive for tuberculosis via the QuantiFERON-TB Gold (QFT) interferon gamma release assay after completing a second four-month treatment with rifampin. He had a past medical history of hyperlipidemia and hypertriglyceridemia but no symptoms for tuberculosis or CAD. After several investigations, it was eventually discovered that the patient had severe stenosis in his left anterior descending (LAD) artery (Figure 1). Initially, low-dose chest CT was done to assess for evidence of tuberculosis and came back negative. However, it was noted that there was coronary atherosclerosis around the epicardium, which was unusual for a man of his age (Figure 2). Assessment for CAD is not usually done with low-dose chest CT and it is typically an incidental finding, as in this case [4]. Further investigation found a highly elevated coronary artery calcium score of 121.2, placing him in the 98th percentile for the same age group as per the radiology report. Following this finding, a CT angiogram (CTA) was done two weeks later and found stenosis of the LAD (Figure 1). The CTA was analyzed with HeartFlow® and found 99% stenosis of the middle LAD right after the diagonal bifurcation (Figure 3).

Approximately two weeks after the abnormal CTA findings, a percutaneous transluminal coronary angioplasty (PTCA) was done with a drug-eluting stent (DES) being placed in the middle LAD after the diagonal bifurcation which had the 99% stenosis (Figure 3). The first diagonal branch of the LAD was found to have 50% proximal stenosis. The proximal and distal LAD were normal. The left circumflex (LCX) artery and right coronary artery (RCA) were also found to be normal. 

There were no complications, and the patient was discharged on dual antiplatelet therapy (DAPT) consisting of aspirin 81 mg orally once a day and brilinta 90 mg orally twice daily. The patient is also currently on fenofibrate 145 mg orally once a day and atorvastatin 10 mg once a day. He reported feeling some fatigue during his primary care follow-up, which is likely myopathy due to being on a fibrate and statin concurrently, but is tolerating it and continues to be compliant. He is also taking ergocalciferol 1.25 mg (50,000 UT) capsule orally once a week.

## Discussion

The median age of CAD diagnosis is 65 years in men, and typically occurs after an MI. Over 15 million Americans currently live with CAD. Globally, almost 20 million deaths were attributed to the disease in 2020, representing 32% of all global deaths [1-3]. It is important to discuss that while mortality due to CAD is decreasing, global incidence is increasing [4]. This is partly due to an aging population in many Western countries and younger people being diagnosed with CAD in developing countries due to the adoption of Western lifestyles and diets as well as a high incidence of men who smoke [4]. This puts a strain on resources, making managing heart disease important from a preventative standpoint [4].

CAD, when asymptomatic and in younger patients, can be particularly challenging to catch and is usually found incidentally [5]. While the mean age of people with CAD is 65 years old, it is no longer a exclusively disease of late adulthood, as between 1995-2014, 30% of all acute MIs were in people aged 35-54 years old [6]. Given that it remains the number one cause of mortality globally, and mortality is directly associated with how early it is detected, we believe it is pertinent to re-examine how and when to screen for it [1,5]. In fact, it has been found that early detection leads to 50% reduction in mortality in CAD [7]. We have known for almost two decades that fatty streaks can be found in teenagers, and we should continue to expect a rise in CAD appearing in a younger patient population [5]. In addition to routine blood work investigating cholesterol, blood sugar levels, and other markers, one potential method is to implement screening with calcium scores, which is a cost-effective way to identify individuals who may need intervention, be it medically with statins and aspirin or, if necessary, more invasive procedures such as coronary angiograms [8]. Recent studies using calcium scores have shown that anywhere from 10-34% in adults under 40 have atherosclerosis [9]. If up to one-third of young adults have measurable plaque build up and it can be detected cheaply and safely, this allows physicians to plan and engage in preventative care. A systematic review found that patients with certain comorbidities such as diabetes, hypertension, smoking, or dyslipidemia are more likely to have an atypical presentation of MI in their fifth decade of life, and MI should be suspected by primary-care physicians or in the emergency department if they present with symptoms such as dizziness, shortness of breath, fatigue, or episodes of syncope [10]. This index of suspicion should be low since nearly 33% of patients with atypical presentations of MI are misdiagnosed at first [10]. That, along with findings that up to 34% of young adults under 50 have measurable atherosclerosis, it is not unreasonable to monitor patients at the primary-care level for these symptoms and refer them for calcium scoring if they have pertinent positives of the above mentioned comorbidities with the associated symptoms [9,10].

Sedentary lifestyles as well as high sodium intake are well studied and documented risk factors for CAD [11]. Furthermore, a systematic review by Agrawal et al. found that young South Asian males, such as our patient, are at higher risk of developing CAD at a young age with the most common risk factor for young men being tobacco use, with two-thirds of CAD patients from all South Asian countries smoking tobacco [12]. One study in West Bengal, India, found that 93.8% of CAD patients under 40 years of age consumed tobacco in some form [4]. The second most common risk factor was diabetes with 80.6% of young South Asian patients with CAD having type 2 diabetes mellitus as a pre-existing comorbidity. Diabetes in particular is associated with a 200% increase in CAD risk [13]. Other common risk factors included dyslipidemia, obesity/high BMI, and family history [4]. Interestingly, when diagnosed single vessel disease (SVD) was more common in this demographic than multi-vessel disease [12]. The end result of these risk factors going unaddressed is coronary artery occlusion, as demonstrated in Figures 2 and 3.

Although management of the disease has improved due to prescriptions of statins and aspirin, it remains expensive for both the healthcare system and the patient. One study found that implementing screening protocols using coronary artery calcium scores was superior to current guidelines for men categorized as intermediate risk (defined as a calcium score between 101-400, which our patient would fall into) [14]. The authors of this study believe that calcium scores can be used to aid in deciding whether or not to prescribe statins. For example, a physician may decide that a younger adult with a high calcium score is in need of a statin earlier than current guidelines suggest based on the objective evidence from the calcium score, and the inverse may also be true where an older adult with a low calcium score may not need statin therapy or may require less aggressive therapy [14]. Additionally, calcium screening in men under 50 with a family history of CAD is more cost-effective than using statin therapy for those with subclinical disease, which was defined as a calcium score ≥ 100 without symptoms [15]. All of this aids in potentially better and more targeted patient care as well as being cost-effective for patients. Furthermore, a prospective randomized control trial found that utilizing calcium scores reduced healthcare costs and testing down the line [16]. Hospitalizations from coronary disease costs the Canadian system $1.7 billion annually [4]. In the United States, patients on average incur an additional annual cost of about $30,000 after being diagnosed with the condition, cumulatively costing the country over $200 billion annually [2,4]. These costs are only going to grow as the population ages and younger people are diagnosed due to the modifiable risk factors discussed above. This will put a strain on healthcare resources and contribute to physician and healthcare worker burnout, which is negatively associated with healthcare outcomes [4,17].

It is imperative that further research is done on effective ways of encouraging positive behavioral changes in the population and re-assessment of how we screen for CAD and at what age [17]. Coronary calcium scoring has come down in cost in recent years and now typically costs less than $150 out of pocket [14-16]. This, measured against decades of potentially unnecessary medication or costly medical intervention in someone who presents to the emergency department with an MI and without having been screened, makes implementing coronary calcium scores financial feasible and clinically justifiable. All of this leads us to contend that while prevention is preferred, early detection is paramount if behavioral modifications are ineffective. 

## Conclusions

Many causes of CAD are due to modifiable risk factors such as diet, type 2 diabetes mellitus, smoking, and activity levels. It is becoming increasingly common for people 40 years old and younger to develop this disease, making prevention an important target. This is significant given that CAD remains the leading cause of mortality worldwide. Additionally, while CAD-associated mortality is decreasing, especially in developed countries, CAD is still responsible for almost one-third of total deaths globally. As this stops being exclusively a disease of late adulthood, it is becoming increasingly important for us to find ways to screen and detect atherosclerosis that can become CAD. Several studies have shown that implementing the use of coronary calcium scores is a cost-effective method to screen patients, allowing for earlier treatment and thus reducing downstream costs and intervention which would lessen the burden on the healthcare system and improve the patient's quality of life.

## References

[1] (2024). Cardiovascular diseases (CVDs). https://www.who.int/news-room/fact-sheets/detail/cardiovascular-diseases-(cvds).

[2] Shahjehan RD, Sharma S, Bhutta BS (2023). Coronary artery disease.

[3] Benjamin EJ, Blaha MJ, Chiuve SE (2017). Heart disease and stroke statistics-2017 update: a report from the American Heart Association. Circulation.

[4] Bauersachs R, Zeymer U, Brière JB, Marre C, Bowrin K, Huelsebeck M (2019). Burden of coronary artery disease and peripheral artery disease: a literature review. Cardiovasc Ther.

[5] Mendoza DP, Kako B, Digumarthy SR, Shepard JO, Little BP (2020). Impact of significant coronary artery calcification reported on low-dose computed tomography lung cancer screening. J Thorac Imaging.

[6] Arora S, Stouffer GA, Kucharska-Newton AM (2019). Twenty year trends and sex differences in young adults hospitalized with acute myocardial infarction. Circulation.

[7] Pakdaman MN, Rozanski A, Berman DS (2017). Incidental coronary calcifications on routine chest CT: clinical implications. Trends Cardiovasc Med.

[8] Di Cesare M, Perel P, Taylor S (2024). The heart of the world. Glob Heart.

[9] Michos ED, Choi AD (2019). Coronary artery disease in young adults: a hard lesson but a good teacher. J Am Coll Cardiol.

[10] Khan IA, Karim HM, Panda CK, Ahmed G, Nayak S (2023). Atypical presentations of myocardial infarction: a systematic review of case reports. Cureus.

[11] Agrawal A, Lamichhane P, Eghbali M (2023). Risk factors, lab parameters, angiographic characteristics and outcomes of coronary artery disease in young South Asian patients: a systematic review. J Int Med Res.

[12] Sau S, Sau S, Mukherjee L (2018). Profile of myocardial infarction in young patient of age 40 years or below in a tertiary care hospital mainly tribal based population. JMSCR.

[13] Saraste A, Knuuti J, Bax J (2023). Screening for coronary artery disease in patients with diabetes. Curr Cardiol Rep.

[14] Hecht HS (2015). Coronary artery calcium scanning: past, present, and future. JACC Cardiovasc Imaging.

[15] Venkataraman P, Kawakami H, Huynh Q (2021). Cost-effectiveness of coronary artery calcium scoring in people with a family history of coronary disease. JACC Cardiovasc Imaging.

[16] Rozanski A, Gransar H, Shaw LJ (2011). Impact of coronary artery calcium scanning on coronary risk factors and downstream testing the EISNER (Early Identification of Subclinical Atherosclerosis by Noninvasive Imaging Research) prospective randomized trial. J Am Coll Cardiol.

[17] Nasir K, Cainzos-Achirica M (2021). Role of coronary artery calcium score in the primary prevention of cardiovascular disease. BMJ.

